# Editorial Notes

**Published:** 1915-12

**Authors:** 


### Editorial Notes.

**Lord Mayor of Bristol.**

To the Lord Mayor of Bristol, Dr. Barclay J. Baron, we offer our congratulations and best wishes for a successful tenure of office. It is many years since the chief magistracy has been held by a medical practitioner in this city, and no one could be better qualified to fulfil its great responsibilities at this time.

**Second Southern General Hospital.**

In our last issue we published the list of those holding commissions on the *en suite* Staff of this Hospital. Since that time many changes have occurred and newly-commissioned officers have been added to the Staff to take the place of those officers who have accepted appointments in the Expeditionary Forces, viz. Captain Hey Groves, now at Alexandria, Captain Fortescue-Brickdale, at Havre. Captain Carey Coombs is attached to No. 32 General Hospital. The following have been added to the Staff: Captain J. A. Nixon, Captain J. R. Charles, Captain F. R. Walters, Captain Rendle Short, Captain A. L. Flemming, Captain Mackenzie, Captain Irving.

Major P. Watson-Williams (Bristol), Dr. Barclay J. Baron (Bristol) and Major C. S. Ridout (Portsmouth) have been appointed by the War Office as Honorary Consultants for Diseases of the Ear, Nose and Throat to Military Hospitals in the Southern Command.

Miss Baillie, Matron of the Royal Infirmary, and in charge of the Red Cross Nursing Staff of the Second Southern General Hospital, has been awarded the Royal Red Cross in recognition of her successful and untiring labours in organising this important side of the Hospital work. We offer her our cordial congratulations.

The work of the Second Southern General Hospital, under the command of Lieut.-Col. Bush, C.M.G., has been greatly extended by the addition of many beds at the Southmead Hospital and by the conversion of the Fishponds Asylum into the Beaufort War Hospital, under the command of Lieut.-Col. Blachford. It is our intention to give in our next issue further notice of this and other matters, for which it is now impossible to find adequate space.

Temporary Lieut. John Wesley Gilbert, R.A.M.C. (attached 9th Brigade, R.G.A.), who has been awarded the Military Cross for conspicuous gallantry and devotion to duty near Ypres, on December 29th, 1915, is the son of the late Mr. James Cornwall Gilbert and Mrs. Gilbert, of 8 Broadway Road, Bishopston, Bristol. The official record of his action is given in the following words: "After three attempts he succeeded in entering a farm, which was being heavily shelled with gas and other shells, and rendered valuable assistance to the wounded infantry who were billeted there."

Dr. Gilbert, who is twenty-four years of age, carried out his medical studies at Bristol University and the General Hospital. He joined the R.A.M.C. in February, 1915, and for five months was operating in a base hospital at Boulogne, later proceeding to Ypres.

**Notes on the Representative Meeting of the British Medical Association.**

On July 23rd and 24th a short but important meeting was held. A large part of the Insurance difficulties had been thrashed out at the conference of the Local Medical and Panel Committees, voiced by their deputation to the Commissioners. We need not discuss the excuses made by the latter for the delays which have caused so much irritation. The Council issued a reply to the attack made by the Bristol Insurance Committee on the sixpence now paid to medical men for domiciliary treatment of tuberculosis, about which it should be remembered that the Bristol doctors proposed and actually worked a cheaper and better method of attendance, and the Government offered them this sixpence as a make-weight and part of their bargain for general work. Indeed, much of the tuberculosis work done for it never appears in the returns at all, and but for the panel doctors the earliest cases would often never be discovered.

New rules of procedure in ethical cases have been elaborated under legal advice to safeguard the Association as far as possible. Again and again it has been involved in expensive and dangerous lawsuits through the ill-advised action of local branches, and the safety of its funds has been imperilled by legal defects in the old rules. In future no other rules will be recognised.

It is a matter of congratulation that the Association has been recognised in at least three important directions as a mouthpiece of the profession.

First, the Head of the Army Medical Service applied to it for help in recruiting medical officers. From this sprang the census or register it compiled of men able to offer their services, and in turn it made representations to the War

Office as to the grievances of men who did take up service. A War Emergency Committee was formed to organise the profession, and to deal with matters connected with the war. Sir W. Osler, Sir Clifford Allbutt, Dr. A. E. Shipley, Mr. Verrall and other members of the Association were placed on it, with power to co-opt representatives of the Universities and licensing bodies. They appealed strongly for volunteers.

It appeared that the Bristol and Weston district had already supplied ninety whole-time men for military service, beside those attached to the Base Hospitals of 3,080 beds existing here, and twenty-eight more men were asked for. Great as the strain was, volunteers responded in a surprising manner, and twenty-eight more men left their practices. Of the 275 medical men living in Bristol and Clifton alone, 111, *i.e.* about 45 per cent. of those actually engaged in private practice, have put themselves entirely at the disposal of the State, eighty-four are serving with the colours and twenty-seven at the Base Hospitals. A large part of the remainder are also acting as Civil Surgeons to the troops.

The Appeal of the Bristol Committee to the public not to desert their own medical men after serving in the war was taken up by the Central Committee and issued in substance to the whole kingdom.

In another direction the Association also won recognition. Some of the leaders of the English and Welsh Friendly Societies have at last met us in a harmonious conference on the old and bitterly-fought question of their Medical Institutes. The change from their former determination to crush the Association to the present atmosphere of reasonable discussion is very great. The Representative Meeting in consequence agreed to recognise existing institutions in Wales, under Clause 15 (4) of the Act, which conform to a certain standard. The negotiations are by no means finished, and we can only say that both sides acknowledge the need of a mutual understanding. The Societies accepted at once some of our positions, and were ready to give up all attempts to make a profit out of the work of the institute doctors, to satisfy us that the funds are not used to lower the price of attendance on dependents, and even on certain conditions to do their best to prevent other institutes or branches of existing ones from being started.

Whether a final agreement is reached or no, we cannot but be thankful that our opponents are getting to understand our point of view, and have shown themselves such able and reasonable men.

Again, the Association has taken action with the Bar Council and the Incorporated Law Society in forming a joint committee to consider important difficulties in the law on insanity. Here, too, agreement was attained with them on old-standing troubles, such as the definition of insanity, the question of uncontrollable impulse, and the position of prisoners found "guilty, but insane." This paves the way for important changes in the law in future. On the other hand, the Representative Meeting made a strong protest, in which they had the support of the Royal College of Physicians, against an attack now being made on professional secrecy. The Association had sent a deputation to the Lord Chief Justice, the Attorney-General, the Public Prosecutor and other officials on May 3rd last. It then appeared that the law officers of the Crown were desirous that medical men should give information to the police when they learn in the course of their attendance upon a patient who is likely to die that an attempt at producing abortion has been made upon her by a third party. This means that they shall disclose without the patient's consent the matter revealed to them, and even go out of their way to publish it.

No indemnity in law is even suggested for a doctor who thus casts aside his obligation to hold secret what his patient tells him, should the patient recover and bring an action. Nor do the law officials seem to trouble about the lives which would be lost if women were deterred from calling in a doctor by the knowledge that he would inform against them and their accomplices. It is our clear duty to do everything we can as citizens to bring the criminals to book, but we cannot use for that purpose information given us to enable us to save life under the seal of professional secrecy.

As things stand we are already unfairly treated, for the lawyers have complete protection as to statements made to them in their professional capacity by clients, but no such privilege is given to medical men when questioned in court, though it is allowed in the French and other codes.

"Yes," said an old judge to his doctor, "if I had been on the Bench the other day I should have done my best to make you disclose what the patient confessed to you, but," he added, "I should have changed my doctor if you did."

The Notification of Births Act is another instance of the present tendency to force doctors to reveal what they see and hear in their professional work. We may heartily support the Act as a means of obtaining proper care for infants, but it should be compulsory on parents and by-standers, not on doctors and midwives, who come in simply to save life. Otherwise people will do without them and risk the consequences. However, it may be said in defence that the Act does not compel the doctor to do anything at all except to see that someone else—the proper person—does it. The meeting registered its protest against this clause of the Act.

In the Report of the Council among other matters are the very satisfactory recommendations of the *Select Committee on Patent Medicines* appointed by the Government.

The Association has the credit of collecting and submitting much valuable evidence to the Committee.

It recommends that the Local Government Board should have the administration of the law as to secret and patent medicines, keep a register of makers and importers, and institute prosecutions against offenders in a specially constituted court. Another recommendation is that a statement of the exact ingredients of every secret remedy is to be deposited privately with the Board, and the article tested shall be for comparison from time to time. The amount of alcohol contained is to be exhibited on the label of every bottle. No advertisement, or sale for private use, of remedies purporting to cure paralysis, cancer, phthisis, epilepsy and similar diseases, and no advertisements of sexual remedies or of any with the suggestion that they may be used as abortifacients is to be allowed. Various fraudulent practices, such as the use of fictitious testimonials, are also penalised.

The war has failed to stop the flow of schemes for spending the funds of the Association. Though at the meetings of every other society and Local Authority economy is the order of the day, endless proposals for the payment of representatives and for costly new committees were showered upon us. Most of them were promptly defeated, but their proposal at this time shows a strange narrowness of view. Though our finances have temporarily improved, the Association is saddled with a huge debt which it is only now beginning to pay off. The interest on this will be higher in future, and in the hard times after the war our rents may fall, and subscriptions may have to be lowered if the membership is to be kept up. The wages bill has already risen, and if a spirit of vigorous retrenchment does not appear the Association finances may soon be worse than ever.

**Sickness Insurance.**

MEMBERS of our profession, more especially, are facing many dangers every day of their lives, and though constant contact with danger produces a feeling of immunity, most doctors nowadays insure themselves against the risk of illness or breakdown as the result of their professional labours.

Medical men are particularly well placed in this respect by having a Society of their own to protect them. Run on the soundest actuarial principles, and on the most economical lines, the Society has attained considerable dimensions. Its income is now over £35,000 per annum, and its invested funds £270,000.

The form of contract is permanent till age 65, and cannot be discontinued before that date, except in the case of fraud, non-payment of premium, or permanent residence abroad. Full Sickness Pay is payable for the first twenty-six weeks' incapacity, and half the full amount for the remainder of the illness. Every kind of sickness and accident is covered, and the premium payable is lower than that of any Company transacting similar business—entirely owing to the fact that no agents are employed and no commission paid. In fact, the management expenses are limited to 10 per cent. of the premium income.

The Committee of Management is composed of medical men, whose services are gratuitous. The members of this Society are therefore assured of more sympathetic treatment than they might obtain from a Board of Directors of a Company with shareholders, who naturally look to the dividends of their concern as an essential factor. All the profits of the Society belong to the members, and some £21,000 has been returned to them in this form.

Members of the medical profession would do well to apply to their own Society for Sickness Insurance rates before accepting those of other Companies (who charge in some cases at least 25 per cent.) more on account of their heavier management expenses. For all information please apply to Mr. Bertram Sutton, Secretary, Medical Sickness and Accident Society, 300 High Holborn, London, W.C.

A BI-CENTENARY in any business is an epoch worthy of congratulations, and these we offer to the Plough Court Pharmacy of Allen and Hanbury, which celebrated the event on December 18th last. The business was founded by Silvanus Bevan in 1715, the Allens and Hanburys coming into the firm now known by their names about the middle of the nineteenth century. As about two hundred of those connected with the firm are away serving their country, the foregathering in honour of the event and for the purpose of presentations to members of the directorate, though largely attended, was less complete than it would have been under happier circumstances.

